# The Significance of Asthma Follow-Up Consultations for Adherence to Asthma Medication, Asthma Medication Beliefs, and Asthma Control

**DOI:** 10.1155/2015/139070

**Published:** 2015-12-07

**Authors:** Malin Axelsson, Linda Ekerljung, Bo Lundbäck

**Affiliations:** ^1^Department of Care Science, Faculty of Health and Society, Malmö University, Jan Waldenströms Gata 25, 20506 Malmö, Sweden; ^2^Krefting Research Centre, Institute of Medicine, Internal Medicine and Clinical Nutrition, Sahlgrenska Academy, University of Gothenburg, Box 424, 405 30 Gothenburg, Sweden

## Abstract

*Objective*. The aim was to investigate adherence to asthma medication treatment, medication beliefs, and asthma control in relation to asthma follow-up consultations in asthmatics in the general population. A further aim was to describe associations between adherence, medication beliefs, and asthma control.* Method*. In the population-based West Sweden Asthma Study, data allowing calculation of adherence for 4.5 years based on pharmacy records were obtained from 165 adult asthmatics. Additional data were collected through questionnaires and structured interviews.* Results*. The mean adherence value for filled prescriptions for regular asthma medication was 68% (median 55.3%) but varied over the year under study. Adherence to combination inhalers with corticosteroids and long-acting beta^2^ agonists was higher than adherence to single inhalers with corticosteroids only. More than one-third of participants reported not having seen an asthma nurse or physician for several years. Regular asthma follow-up consultations were associated with both higher adherence and the belief that asthma medication was necessary but were not associated with asthma control.* Conclusions*. Adherence to asthma medication treatment was low and varied over the year under study. The current study suggests that quality improvements in asthma care are needed if adherence to asthma medication is to be improved.

## 1. Introduction

Previous studies have shown that there is considerable room for improvement in adherence to asthma medication treatment [1–3]. Because poor adherence may reduce the likelihood of achieving and maintaining good asthma control, it is important that adherence to prescribed asthma medication be addressed by the health-care provider during regular follow-up consultations [4]. Adherence may be regarded as a multifaceted behaviour that is influenced by a variety of factors, one of which is the health-care provider [5]. Asthma clinics led by specially trained asthma nurses have been shown to be effective in asthma management, for instance, in relation to adherence [6]. Moreover, the interaction between the health-care professional and the patient regarding joint treatment decisions, taken by the asthma patient and the clinician together, seems to have a positive effect on adherence as well [7]. Another factor affecting adherence is beliefs about the asthma medication [8–11]. Individuals with asthma who, for instance, believe that their medication is necessary for their present and future health or that it prevents exacerbation of their disease are more likely to be adherent. In contrast, individuals who are concerned about their asthma medication are more inclined to deviate from the prescribed treatment [8–10]. It has also been reported that individuals with uncontrolled asthma tend to be sceptical about their asthma medication, which may cause them to choose symptom management strategies other than the medication [12].

Asthma is often studied in clinical settings, where the population of patients does not necessarily reflect individuals with asthma in general. Additionally, few studies have addressed treatment adherence, medication beliefs, and asthma control in relation to reported asthma follow-up consultations in individuals with asthma in the general population. It could be hypothesised that asthma follow-up consultations may have a positive influence on adherence to asthma medication treatment and medication beliefs and that adherence, medication beliefs, and asthma control would be associated. The aim of the present study was to investigate adherence to asthma medication treatment, medication beliefs, and asthma control in relation to asthma follow-up consultations in individuals with asthma in the general population. A further aim was to describe associations between adherence, medication beliefs, and asthma control.

## 2. Materials and Methods

### 2.1. Study Sample

**Figure 1:**
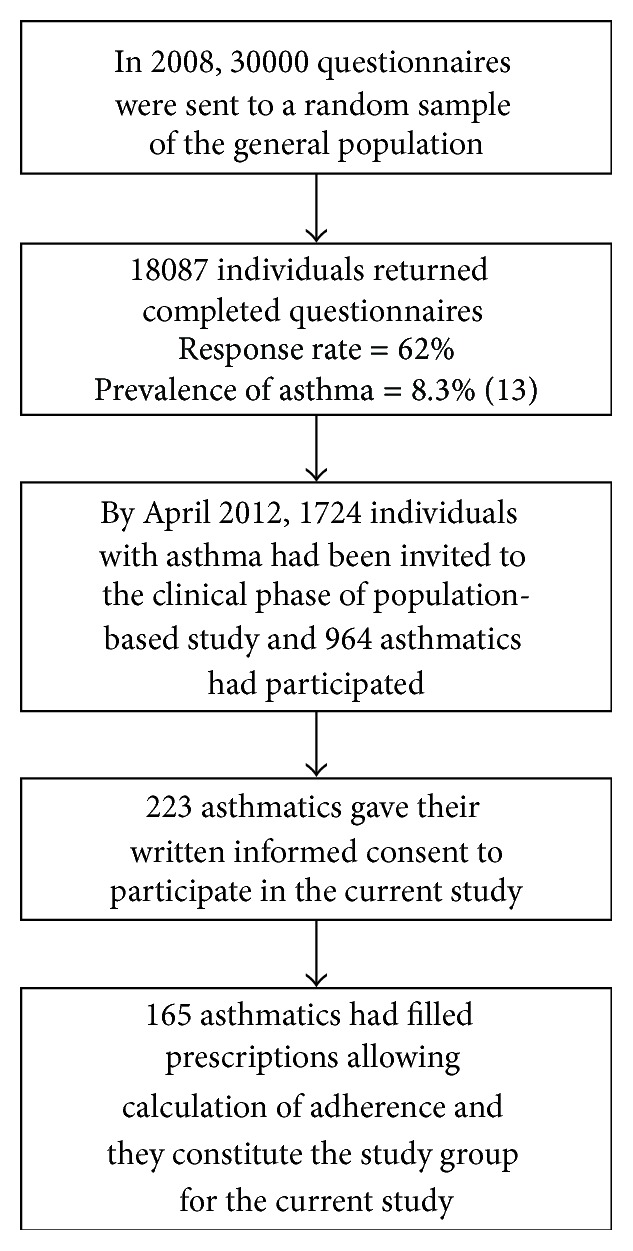
Sampling procedure.

The sample was derived from the population-based West Sweden Asthma Study (WSAS), which has been thoroughly described elsewhere [13]. The sampling procedure is illustrated in Figure 1. Briefly, 30,000 inhabitants selected at random were invited to complete postal questionnaires including questions about symptoms, diseases [13], exposures, and occupation [14]. Of the 18,087 returned completed questionnaires, 3524 participants (1724 of whom were asthmatics) were invited to take part in the clinical phase of the study. By April 2012, 964 adult asthmatics had participated in the clinical study and 223 had given their written informed consent, allowing us to collect data concerning them from the drug registry maintained by the National Board of Health and Welfare. The Regional Research Ethics Board at the University of Gothenburg approved the study.

### 2.2. Data Collection

Calculations of adherence were based on data from the drug registry on asthma medication prescribed for regular use. First, calculations for all regular asthma medications were made. Then separate calculations were made for regular prescriptions for single inhalers with corticosteroids and for combination inhalers with corticosteroids and long-acting beta^2^ agonists. The observation period started on each participant's first date of prescription fill after 2008-01-01 and ended on each participant's last date of prescription fill before 2012-06-30. Defined daily dose (DDD) is “*the assumed average maintenance dose per day for a drug used for its main indication in adults*” [15, p. 22]. DDD is a unit of measurement calculated by the World Health Organization to enable comparisons between studies focused on, for example, medication utilization [15] and was therefore used in the current study. The DDDs for the last filled prescription were excluded from the calculations. Adherence was calculated as the sum of all DDDs divided by the observation period and multiplied by 100, giving a percentage measure of adherence.

The Beliefs about Medicines Questionnaire (BMQ) consists of 10 items, scaled 1–5, and was used to assess beliefs about prescribed asthma medications. Five items were used to measure a person's beliefs about the necessity of asthma medication and five items to measure concerns about asthma medication [16]. Cronbach's alphas in the current study were 0.884 for the necessity scale and 0.798 for the concern scale.

The Asthma Control Test (ACT) consists of five items, scaled from 1 to 5. A cut-off point of ≤19 indicates poorly controlled asthma, and scores of 20 points or more indicate controlled asthma [17]. Cronbach's alpha in the current study was 0.730.

Data on asthma follow-up consultations were gathered through structured interviews during the clinical phase of the WSAS.

### 2.3. Analysis

Percentages, means, standard deviations (SD), medians, and interquartile ranges (IQR) were used to describe the study sample. Pearson's correlation coefficients (*r*) were used to explore associations between adherence, medication beliefs, and asthma control. Student's *t*-tests were used to determine differences between subgroups in the study sample.

## 3. Results

### 3.1. Study Participants

Of the 223 participants, 165 (109 women (66%) and 56 men (34%); mean age 49.65 years (SD 15.64)) had filled prescriptions for regular asthma medication, which enabled calculation of adherence. Of these 165 participants, 36% reported not having seen an asthma nurse or physician for several years, and 36% reported that they usually saw an asthma nurse or physician once a year or more often. More than half of the participants received their prescriptions for asthma medication when visiting physicians for reasons other than asthma. Most participants perceived their asthma to be controlled (mean 20.50, SD 3.60) (Table 1).

### 3.2. Adherence in relation to Prescribed Regular Asthma Medication

**Figure 2:**
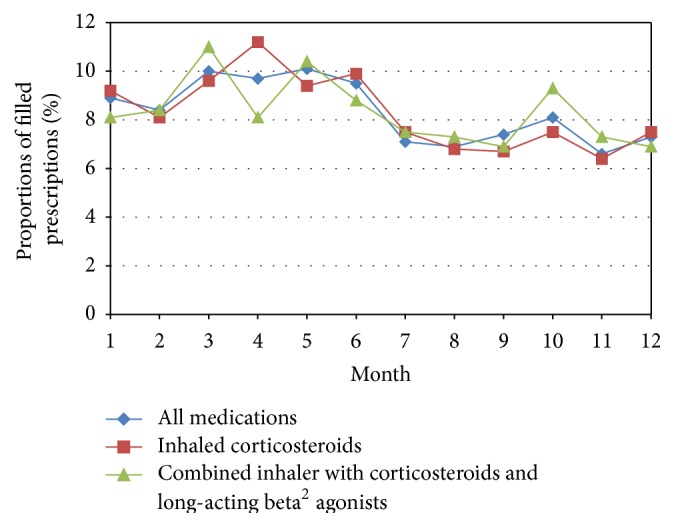
Proportions of filled prescriptions distributed over months of the year. If the filling of prescriptions were evenly distributed over the year, all months would have a filling adherence value of 8.3% (1/12).

**Figure 3:**
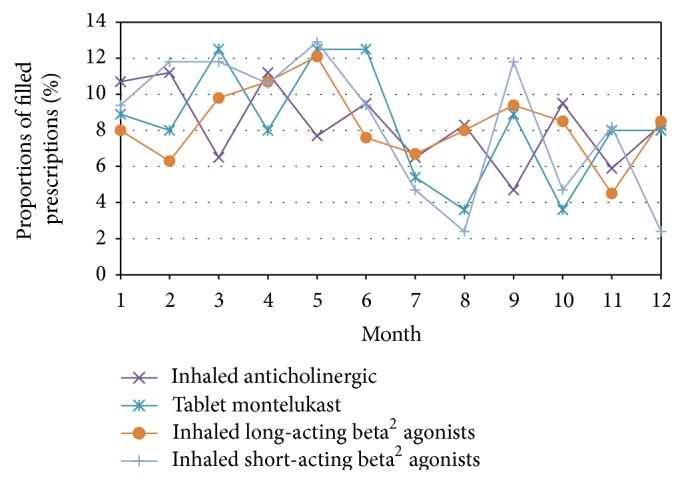
Proportions of filled prescriptions distributed over months of the year. If the filling of prescriptions were evenly distributed over the year, all months would have a filling adherence value of 8.3% (1/12).

The average adherence value was estimated to be 68% (SD 57.80, median value 55.3%, and IQR 73.8), based on filled prescriptions for all asthma medications prescribed for regular use. A comparison of adherence values between men and women showed no gender difference. The average adherence value for inhaled corticosteroids only was 49% (SD 38.67, median value 39.9%, and IQR 54.2). The average adherence value for combination inhalers was 67% (SD 39.18, median value 62.2%, and IQR 55.4). Figures 2 and 3 show patterns of filled prescriptions on a yearly basis. If the filling of prescriptions were evenly distributed over the year, all months would have a filling adherence value of 8.3% (1/12). For example, Figure 2 shows that the proportions of filled prescriptions were higher during the spring and lower during the autumn/winter.

### 3.3. Adherence in relation to Beliefs about Medications and Asthma Control

There was a positive association between the belief that the asthma medication was necessary and adherence (*r* = 0.210, *p* < 0.012), indicating that adherence was higher among individuals believing that their medication was necessary. There was a negative association between the belief that the asthma medication was necessary and asthma control (*r* = −0.174, *p* < 0.045), indicating that individuals with better asthma control perceived their asthma medication as less necessary. No significant associations were found between concerns about the asthma medication and adherence or asthma control. No association between asthma control and adherence was found.

### 3.4. Adherence, Medication Beliefs, and Asthma Control in relation to Asthma Follow-Up Consultations

Both higher adherence and higher ratings on the necessity scale were reported among participants who visited their asthma nurse or physician at least once a year and monitored their asthma using a peak-flow metre. Moreover, lower adherence was found among participants who had not seen an asthma nurse or physician for several years. Lower adherence was also reported among participants who had received prescriptions for asthma medication when visiting physicians for reasons other than asthma (Table 2). No differences were observed regarding concerns about asthma medication and asthma follow-up consultations. Additionally, no differences were observed regarding asthma control and asthma follow-up consultations (Table 2).

## 4. Discussion

Based on the current findings, poor adherence to preventive medication was common among asthmatics in the general population. However, adherence to combination inhalers was somewhat higher compared to single inhalers with corticosteroids only. Asthma medication prescriptions were not refilled regularly throughout the year. The belief that the asthma medication was a necessity was associated with better adherence but no associations between asthma control and adherence were observed. Asthma follow-up consultations at least once a year and using a peak-expiratory-flow metre were associated with better adherence and believing that asthma medication was a necessity.

In line with previous adherence research on individuals with asthma, the current study showed that adherence to prescribed medication treatment could be improved [1–3]. Of further importance is the finding that adherence was higher among participants who had yearly asthma follow-ups, because it indicates that asthma nurses and physicians play a role in promoting adherence. Scheduling regular follow-up visits with asthma patients and reviewing their adherence behaviour would most likely increase adherence. Regular visits may also prevent patients from receiving asthma medication prescriptions during other health-care visits, a phenomenon that was associated with lower adherence in the current study. However, the higher adherence found among participants who had yearly follow-up consultations could perhaps also be explained by a higher level of motivation [18]. Another explanation could be disease severity but low adherence in individuals with difficult asthma has also been reported [19]. In the current study, no difference in reported asthma control could be related to the participants' health-care utilization suggesting that disease severity did not explain the better adherence found among participants who had regular asthma follow-up consultations. In pediatric asthma care regular consultations with health-care professionals have been associated with improved adherence [20], which further supports the current findings arguing that regular asthma follow-ups are important if adherence to asthma medication is to be improved.

It is recommended in asthma guidelines that individuals with asthma be reviewed regularly in order to assess asthma control as well as response and adherence to prescribed medication treatment [4]. However, a large proportion of participants in the current study reported that they had not visited an asthma nurse or physician for several years and that they had received their prescriptions for asthma medication when visiting physicians for reasons other than their asthma. These findings indicate that the asthma care falls short, which is in line with previous research showing that there is a need for quality improvement regarding clinical evaluation and follow-up consultations for individuals with asthma [21]. A possible explanation for the overall low adherence in the current study may be the absence of regular asthma follow-up consultations during which a partnership between the individual and the health-care provider could be established. Such a partnership is crucial as it enables the individual with asthma to acquire knowledge, confidence, and skills necessary for an effective asthma management [4]. Furthermore, regular follow-up consultations allow joint treatment decisions, which are known to have a positive impact on adherence to asthma medication treatment [7].

Regarding the influence of treatment-related factors on adherence, it has been argued that efforts on the part of pharmaceutical companies to develop medication treatments intended to facilitate adherence, such as combination therapies, do not appear to be reflected in increased adherence figures [22]. However, in the current study, adherence to combination inhalers was higher among participants prescribed combination inhalers, which both is in line with [23] and contradicts [24] previous studies. The higher adherence values among participants prescribed a combination inhaler could perhaps also be explained by their having more severe asthma, which was not investigated in the current study.

We could show that adherence peaked during spring and reached a minimum in early autumn. There could be different explanations for this finding but two reasonable speculations for this uneven distribution may be the pollen season and that asthma medication use is symptom-driven. The variability of asthma symptoms is a well-known obstacle to achieving satisfactory adherence to asthma medication treatment [25]. Therefore, having an asthma checkup with one's asthma nurse or physician when adherence is lowest during early autumn may be one method of improving adherence. Another speculation for the drop in adherence after pollen season could be according to advice from their physician or asthma nurse to use the asthma medication only in periods of symptoms, which is inconsistent with asthma guidelines if the patient has persistent asthma. However, previous research has shown that the prescribing of asthma medication does not always adhere to national guidelines [26, 27], which naturally could be reflected in patients' adherence behaviour and thereby explain the present findings.

Inconsistent with previous research, no association between asthma control and adherence was found [28], which was unexpected. Instead, a negative association between beliefs that the asthma medication was necessary and asthma control was found which could be interpreted as the medication was not regarded as necessary when having achieved good asthma control. This finding is consistent with previous research showing that perceived good asthma control is a common reason for not taking the asthma medication [29]. Interestingly, participants who had their asthma reviewed by an asthma nurse or physician once a year or more often reported higher ratings on the necessity scale than participants who did not have yearly consultations. This finding could indicate that beliefs about asthma medication are addressed and concerns resolved by the asthma nurse or physician during the follow-up consultations. Another possible explanation could be that participants with higher necessity beliefs are more likely to have their asthma regularly reviewed. Because beliefs about asthma medication are important determinants of adherence [8–11], the current study suggests that both adherence and beliefs about asthma medication should be addressed during asthma follow-ups.

### 4.1. Methodological Considerations

One weakness of the present study is its somewhat small sample size. A speculation for the nonresponse may be that the nonresponders were not prescribed regular asthma medication treatment. Another speculation could be that it could be perceived as problematic to be controlled in a registry. A potential strength is that the sample was selected from a population-based study, which reflects how adherence patterns are presented at the population level during a longer period of time. A possible shortcoming may be that the participants were not recruited to the current study based on clinical examinations, which could have been an option to verify the diagnoses. Regarding assessments of adherence, there is to date no available gold standard, as every method has its shortcomings [5]. One common method of estimating adherence, both in the research and in clinical settings, is through self-reports. One weakness associated with using self-reports is that the obtained adherence values may be influenced by recall bias or social desirability bias, possibly resulting in falsely high adherence values [30]. Use of data from pharmacy records to obtain adherence values and the fact that the adherence results are based on a rather long observation period of 4.5 years may be seen as strengths. In light of the somewhat small sample size, a larger study is recommended for future research.

## 5. Conclusions

The present study showed that, in individuals with asthma in the general population, adherence to asthma medication was low. Although adherence to combination inhalers was higher compared to a single inhaler with inhaled corticosteroids, it was nonetheless low. Regular asthma follow-ups seemed to have a positive effect on both adherence and belief in the asthma medication, indicating that asthma physicians and nurses are important actors in promoting adherence and positive beliefs about asthma medication treatment. Because adherence in relation to asthma medication varies over the year, one good practice for asthma nurses and physicians would be to always discuss adherence and medication beliefs with patients and to follow up prescribed medication treatment after pollen season, when their asthma symptoms have faded. Because a large proportion of participants reported that they had not visited an asthma nurse or physician for several years, the current study suggests that quality improvements in asthma care are warranted if adherence to asthma medication is to be improved.

## Figures and Tables

**Table 1:** Study sample characteristics (*n* = 165).

Age mean (SD)	49.65 (15.64)

	*N* (%)

Sex	
Men	56 (34)
Women	109 (66)
Asthma control mean (SD)	20.50 (3.60)

	*N* (%)

Regular asthma medication	
Inhaled corticosteroids	106 (64)
Combined inhaler with corticosteroids and long-acting beta^2^ agonists	75 (45)
Inhaled long-acting beta^2^ agonists	42 (25)
Inhaled short-acting beta^2^ agonists	38 (23)
Inhaled anticholinergic	12 (7)
Tablet montelukast	22 (13)
Tablet Bricanyl	3 (2)
Asthma follow-up consultations	
I have not seen an asthma nurse or physician for several years	60 (36)
I usually see an asthma nurse or physician once a year or more often	59 (36)
I usually receive prescriptions for asthma medication when visiting physicians for reasons other than asthma	97 (59)
I have a peak-expiratory-flow metre at home that I use	54 (33)
I have visited a specialist in pulmonology or allergy because of asthma at least once	89 (54)
I have had a lung function test at least once	126 (76)

**Table 2:** Adherence, beliefs of asthma medication, and asthma control in relation to asthma follow-up consultations.

Variables		Adherence		Necessity		Asthma control	
		Mean (SD^a^)	*p* value	Mean (SD^a^)	*p* value	Mean (SD^a^)	*p* value
I have not seen an asthma nurse or physician for several years	Correct Not correct	53.36 (43.43) 71.93 (59.31)	**0.038**	15.80 (5.40) 17.34 (4.95)	0.091	20.49 (3.70) 20.59 (3.47)	0.875

I see an asthma nurse or physician once a year or more often	Correct Not correct	76.35 (51.37) 57.30 (55.0)	**0.034**	18.30 (4.65) 15.69 (5.26)	**0.004**	20.54 (3.75) 20.56 (3.44)	0.983

I usually receive prescriptions for asthma medication when visiting physicians for reasons other than asthma	Correct Not correct	57.40 (47.15) 79.30 (63.46)	**0.017**	16.59 (5.16) 16.97 (5.27)	0.683	20.07 (3.75) 21.31 (3.15)	0.056

I have a peak-expiratory-flow metre at home that I use	Correct Not correct	79.64 (53.74) 56.47 (53.03)	**0.011**	18.40 (3.98) 15.77 (5.54)	**0.004**	20.40 (3.32) 20.64 (3.70)	0.704

I have visited a specialist in pulmonology or allergy because of asthma at least once	Correct Not correct	71.17 (58.62) 55.57 (46.46)	0.079	17.31 (5.29) 16.00 (4.95)	0.144	20.11 (3.77) 21.13 (3.19)	0.104

I have had a lung function test at least once	Correct Not correct	66.67 (55.43) 52.54 (44.23)	0.232	16.97 (5.06) 16.22 (5.52)	0.536	20.41 (3.56) 20.86 (3.51)	0.590

^a^Standard deviation.
